# A national-scale assessment of land use change in peatlands between 1989 and 2020 using Landsat data and Google Earth Engine—a case study of Ireland

**DOI:** 10.1007/s10113-023-02116-0

**Published:** 2023-09-20

**Authors:** Wahaj Habib, John Connolly

**Affiliations:** Discipline of Geography, School of Natural Sciences, Trinity College Dublin, Dublin 2, Ireland

**Keywords:** Blanket bogs, Raised bogs, Land use change, Machine learning, Remote sensing, Google Earth Engine

## Abstract

Over the centuries, anthropogenic pressure has severely impacted peatlands on the European continent. Peatlands cover ~ 21% (1.46 Mha) of Ireland’s land surface, but 85% have been degraded due to management activities (land use). Ireland needs to meet its 2030 climate energy framework targets related to greenhouse gas (GHG) emissions from land use, land use change and forestry, including wetlands. Despite Ireland’s voluntary decision to include peatlands in this system in 2020, information on land use activities and associated GHG emissions from peatlands is lacking. This study strives to fill this information gap by using Landsat (5, 8) data with Google Earth Engine and machine learning to examine and quantify land use on Irish peatlands across three time periods: 1990, 2005 and 2019. Four peatland land use classes were mapped and assessed: industrial peat extraction, forestry, grassland and residual peatland. The overall accuracy of the classification was 86% and 85% for the 2005 and 2019 maps, respectively. The accuracy of the 1990 dataset could not be assessed due to the unavailability of high-resolution reference data. The results indicate that extensive management activities have taken place in peatlands over the past three decades, which may have negative impacts on its ecological integrity and the many ecosystem services provided. By utilising cloud computing, temporal mosaicking and Landsat data, this study developed a robust methodology that overcomes cloud contamination and produces the first peatland land use maps of Ireland with wall-to-wall coverage. This has the potential for regional and global applications, providing maps that could help understand unsustainable management practices on peatlands and the impact on GHG emissions.

## Introduction

Peatlands are one of the most space-efficient carbon stores in the world and play an important role in the global carbon cycle (Limpens et al. 2008; Yu et al. 2011). These ecosystems cover about 3% or 4.23 million km^2^ of global land surface area (Xu et al. 2018) and store approximately 455 to 582 Peta grams of carbon (Gorham 1991; Jackson et al. 2017). For centuries, management-related activities have exerted severe pressure and caused the degradation of peatlands (Tanneberger et al. 2021). Consequently, peatland ecosystems are projected to shift from a carbon sink to a carbon source within this century (Loisel et al. 2021).

Emissions from peatlands where substantial land-use change occurred, e.g. forestry and agriculture, have a greater impact on global land atmospheric Greenhouse Gas (GHG) emissions than previously thought (Ribeiro et al. 2021). Afforestation of natural peatlands and intensive forest harvesting also increase CO_2_ emissions and affect water quality (Jovani-Sancho et al. 2021; Palviainen et al. 2021). Furthermore, drained peatlands used for industrial and domestic peat extraction are conduits of fluvial C and GHG emissions. They are also associated with higher risks of carbon loss through fire (Waddington et al. 2001; Turetsky et al. 2015; Wilson et al. 2015; Tubiello et al. 2016; Regan et al. 2019; Peacock et al. 2021). Tiemeyer et al. (2016) analysed GHG emissions from several bog and fen sites dominated by grasslands and reported high emissions. It is evident that these peatland land uses play a significant role in fluvial C and GHG dynamics.

Legislative changes in the EU’s 2030 Climate Change Framework mean that wetlands are now included in the quantification of GHG emissions and removals from LULUCF (Land Use, Land Use Change Forestry: EU 2018). Additionally, the EU Nature Restoration law aims to restore the degraded ecosystem across the EU (EU 2022), while the EU biodiversity strategy for 2030 (2020/2273(INI)) offers comprehensive guidance on the restoration of wetland ecosystems. On a national scale, the Government of Ireland made two major announcements: (1) the allocation of funding for rehabilitation activities on managed peatlands (Government of Ireland 2020) and (2) to report emissions from managed wetlands, including peatlands (Government of Ireland 2021). Given the increased need to accurately quantify emissions from peatlands, as well as carry out rehabilitation, restoration and rewetting activities, it is necessary to map the spatial and temporal changes in peatland land use.

Despite these requirements, there is a lack of spatial information on land use and land use change within these ecosystems (Fluet-Chouinard et al. 2023). Peatlands are not thoroughly represented in global, regional and national land use and land cover classification maps (Krankina et al. 2008; Pflugmacher et al. 2007; UNEP 2022). This is also evident in the efforts to develop peatland-specific global and regional maps (Xu et al. 2018; Montanarella et al. 2006; Tanneberger et al. 2017), as they are often underrepresented in land cover databases. One of the reasons for this underrepresentation is that peatlands have undergone land use conversion to agriculture and forest, or have been mined and thus the land cover has changed (Connolly 2018). Therefore, it is these land cover types that are represented in land cover maps and not the peatlands which are effectively “hidden” beneath (UNEP 2022). This results in an underestimation of peatland area in land cover maps such as the NLCD (National Land Cover Database: Loveland et al. 2000); the European CORINE (Co-ordinated Information on the Environment: Bossard et al. 2000) land cover dataset and the international land use land cover change dataset by IGBP (Fry et al., 2009). Where they are represented such as in the NLCD or the IGBP land cover maps, it is under the broad classification of wetlands. Similarly, in CORINE land cover, which is one of the principal sources of land cover information in Europe, peatlands are categorised under wetlands and are classified as peat bogs (Bossard et al. 2000). Additionally, the CORINE land cover dataset has low spatial resolution with a minimum mapping unit of 25 ha. In many areas across the globe, peatlands that have undergone land use change still contain a large stock of carbon (Hastie et al. 2022). Consequently, as these land cover maps do not adequately represent peatland extent and thus land use on peatlands, it prompts the need to develop peatland land use-specific maps to track land use change over time.

In Ireland, peatlands account for ~ 21% or 1.46 Mha of the total land surface (Connolly and Holden 2009). However, 85% has already been degraded due to land use activities, such as drainage and subsequent conversion to agriculture, forestry and extraction (domestic/industrial) (Malone and O’Connell 2009; Davies and Forster 2014; Connolly 2018; Fluet-Chouinard et al. 2023). Large areas of peatland are managed by semi-state companies, namely Bord na Móna (BnM) and Coillte. BnM was responsible for industrial peat extraction until 2020 when extraction ceased. Coillte, the semi-state forestry company, has afforested a substantial portion of the peatlands in Ireland (Connolly and Holden 2011). There are also several medium and small private companies still actively involved in peat extraction as well as numerous smaller landowners, who manage peatlands for a variety of land uses such as agriculture, afforestation and peat extraction (Malone and O’Connell 2009). However, the spatial extent of these activities has not been mapped.

The Derived Irish Peat Map version 2 (DIPMv2: Connolly and Holden 2009), was used here to delimit peatland areas and then to track land use change since 1990. Land use maps can be produced using readily available satellite remote sensing data. However, persistent cloud cover is a serious impediment to detecting land use change using remote sensing, especially in Europe and more specifically in Ireland (Tolnai et al. 2016). This is also clear from previous Irish land use and land cover mapping efforts which used remote sensing methods (Walsh et al. 2021; Cawkwell et al. 2018, 2010; Fealy et al. 2009). Cloud cover was also an issue for the Connolly (2018) peatland land use study, which was the first map of land use on peatlands in Ireland and used imagery from 2005/6. In that map, four major land use classes were mapped: industrial peat extraction, grassland, forestry and residual peatland. However, areas along the western seaboard were omitted due to cloud cover. Given that areas contain extensive blanket bogs which contain some of the largest peatlands soil organic carbon stocks in Ireland, it is essential to track and monitor the land use there (Renou-Wilson and Byrne 2015).

The past decade has seen a paradigm shift in remote sensing from change detection to monitoring and tracking change through time (Woodcock et al. 2020). This has been paralleled and facilitated by the development of planetary-scale cloud-computing platforms such as Google Earth Engine (GEE: Gorelick et al. 2017) and the Geo-Microsoft Planetary Computer Program (Lukacz 2022). These platforms provide readily accessible computing capabilities and integrated remote sensing image archives thus facilitating the processing and analysis of earth observation imagery for large spatial scales. Furthermore, there has been a substantial rise in freely accessible remote sensing data sources like Sentinel-2 and Landsat 8 and 9, contributing to a notable increase in remote sensing-based peatland mapping and monitoring (Dronova 2015; Czapiewski and Szumińska 2021). To address the issue of cloud contamination, temporal mosaicking functions within GEE can be employed. Amani et al. (2019) and Mahdianpari et al. (2020) utilised these mosaicking functions in GEE to create cloud-free mosaics using seasonal imagery and successfully performed large-scale wetland mapping with wall-to-wall coverage.

In this study, GEE was used with Landsat data (5 and 8) and a machine learning-based classification algorithm, i.e. random forest to assess, monitor and track land use change on Irish peatlands from 1990, 2005 and 2019. The rationale for choosing these key years is that they relate to baseline data requirements for both the Kyoto Protocol and the Paris Agreement. Additionally, the EU 2030 climate and energy framework aims to reduce (at least 40% cuts) GHG emissions to 1990 levels (UN 1992; EU 2018). The baseline year for quantification of GHG emissions from LULUCF for the Paris Agreement is 2005. Lastly, 2020 had been included for a more recent comparison. To address the issue of cloud cover, a 3-year time period (TP) was selected to facilitate the development of wall-to-wall cloud-free images using the GEE mosaicking functions. These TPs consisted of 1 year before and 1 year after the key years, i.e. TP1 = 1989–1991, TP2 = 2004–2006 and TP3 = 2018–2020. The results demonstrate the evolution of land use on peatlands and its current status (TP3), providing accurate spatial data for informed decision-making in sustainable land use management in peatlands.

## Material and methods

### Study area

This research focuses on Irish peatlands, found within Ireland’s temperate maritime climate, which is characterised by mild winters, cool summers and abundant rainfall throughout the year. The spatial extent of peatlands was delineated using DIPMv2 (Connolly and Holden 2009). This map classifies peatlands into three distinct classes: (i) high-level montane blanket bog, (ii) low-level Atlantic blanket bog and (iii) raised bog. Blanket bogs are characterised by their extensive coverage with a “blanket-like” continuous layer of peat. They represent most of the Irish peatlands; 926,700 ha (65 %) and are present mostly, near low-lying coastal plains and across gentle slopes and mountain plateau. In contrast, raised bogs, known for their dome-shaped peat accumulation, cover ~ 533,300 ha (35%) of the peatlands in Ireland and are situated mostly in the inland areas (midlands) (Feehan and O’Donovan 1996; Connolly and Holden 2009). There is also the presence of minerotrophic peatland, fens which cover approximately 22,180 ha (Foss 2007). These were excluded from Connolly and Holden (2009) work on DIPMv2 and therefore are not considered in this study.

### Satellite image data

Landsat-5 and 8 multitemporal and multispectral optical remote sensing satellite images were used in this study. Six spectral bands were selected for the land use classification: red, green, blue, near infrared and both short-wave infrared. The swath width of the sensor is 185 km and has a spatial resolution of 30 m (USGS 2016). Atmospherically corrected Landsat-5 and Landsat-8 products were used including Tier 1 Surface Reflectance from the TM (Thematic Mapper) and OLI (Operational Land Imager). The atmospheric correction of TM and OLI products is based on the Landsat Ecosystem Disturbance Adaptive Processing System and Landsat-8 Surface Reflectance Code respectively (Masek et al. 2006; Vermote et al. 2016; Foga et al. 2017). It includes snow, water, cloud and shadow masks that are based on the per-pixel saturation mask and CFMask (C Function of Mask) algorithm (Foga et al. 2017). The QA (quality assessment) band is used to represent these masks within the Level-1 data. The CFMask has been validated by Foga et al. (2017) and shown to have the best overall accuracy among different cloud-masking algorithms. Topographic effects can also have an impact on the classification results (Álvarez-Martínez et al. 2018a, b). However, most of the study area did not have significant terrain variations, so topographic corrections were not explicitly considered for this study. The entire study area was covered by 9450 columns and 14,341 rows of Landsat imagery for each TP.

### Methodology

**Fig. 1:**
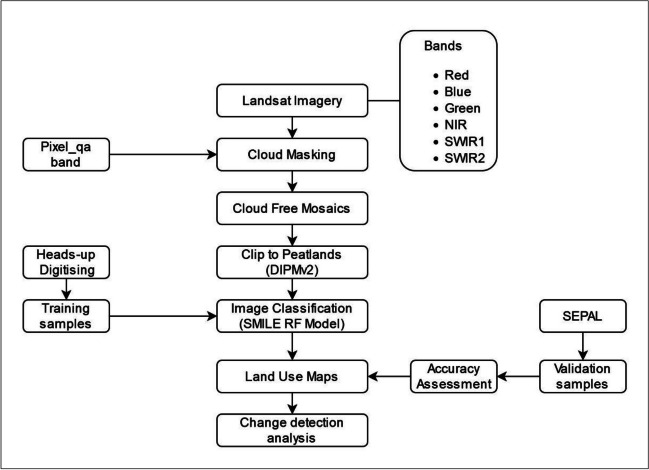
A schematic representation of the data processing workflow based on the processing of the data in the GEE and validation and accuracy assessment using the SEPAL platform. QA (quality assessment), DIPMv2 (Derived Irish Peat Map version 2), SEPAL (System for Earth Observation Data Access, Processing and Analysis for Land Monitoring), NIR (near infrared), SWIR (short-wave infrared), SMILE RF (Statistical Machine Intelligence and Learning Engine Random Forest)

A remote sensing-based image classification approach was used here (Lu and Weng 2007). This included the acquisition and processing of satellite imagery which was conducted in GEE. The imagery was processed to mask out the clouds using the *pixel_qa* band. It was then constrained to the peatland areas in Ireland using the DIPMv2. After defining training areas using heads-up digitisation, a classification of land use was performed using the Statistical Machine Intelligence and Learning Engine (SMILE) random forest algorithm. Accuracy assessment of the results was performed using System for Earth Observation Data Access, Processing and Analysis for Land Monitoring (SEPAL) (Fig. 1).

### Training data

The spatial resolution of the Landsat data (30 m) means that only certain features on the ground can be discriminated. In this case, only four classes could be identified: industrial peat extraction, forestry, grassland and residual peatland (Table 1). These classes are also consistent with the land-use map developed by Connolly (2018) which used a relatively higher spatial resolution (23 m) imagery but did not provide wall-to-wall coverage due to cloud cover contamination along the western seaboard.

**Table 1:** Description of each land use class mapped in the study

Land use class	Description
Industrial	Land that has been subjected to peat extraction, with the peat removed and the land left bare with a thin layer of soil. Managed by BnM with large-scale mechanised peat extraction
Forest	Land covered with trees; afforested areas are mostly covered by evergreen tree species
Grassland	Natural and/or agricultural grassland that is used for pasture or hay, silage and grazing
Residual peatland	Mosaics of revegetated areas (heath, scrub, etc.), exposed peat through domestic peat extraction, remnant peatlands (high bog)

**Fig. 2:**
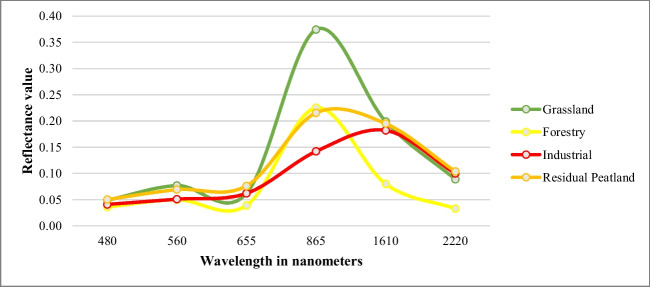
Spectral reflectance of the land use types across the six spectral bands

The training data was derived from randomly distributed polygons digitised by an expert operator using the visual interpretation (Richards 2013) of Landsat imagery available for each TP. Training samples based on these polygons were created for each land use class and each TP in GEE enabling image classification. Figure 2 depicts the spectral reflectance values of the training samples at different wavelengths. There is a significant difference in reflectance values between grassland, forestry and industrial classes at different wavelengths and more noticeable in the NIR region. Residual peatland class, on the other hand, may exhibit some similarities with other land use classes at specific wavelengths, given it includes cutover and dynamic vegetation environment. Nevertheless, the differences in spectral reflectance between all classes are prominently discernible. Hence, the variation observed indicates potential effectiveness in distinguishing the mapped land use types in this study.

### Satellite image processing

To address the issue of persistent cloud cover in Landsat imagery, an efficient cloud removal methodology is crucial in regions where cloudy conditions are prevalent (Amani et al. 2019). Recent studies have shown that a period of 3 years (for a particular season) is optimal to create a cloud-free mosaic for national-scale mapping (Amani et al. 2019; Mahdianpari et al. 2020). Therefore, in this study, GEE was utilised to access imagery with less than 10% cloud cover over each TP to create cloud-free mosaics. It is also pertinent to mention that most peatlands investigated in this research do not experience land use change on short temporal scales. Hence, it was also suitable to use imagery over the entire year as opposed to being constrained to a specific season. This also allowed for the acquisition of wall-to-wall coverage of cloud-free imagery without any gaps created due to the application of cloud masks. The table shows the temporal coverage of images on each TP (Table 2).

**Table 2:** Temporal coverage of the imagery used for each TP. *TM*, Thematic Mapper; *OLI*, Operational Land Imager

Time period (TP)	Spacecraft	Sensor	Temporal selection	Total images
TP-1	Landsat-5	TM	01/01/1989–31/12/1991	387
TP -2	Landsat-5	TM	01/01/2004–31/12/2006	358
TP -3	Landsat-8	OLI	01/01/2018–31/12/2020	500

Based on the selection criterion of temporal (Table 2) and cloud coverage (less than 10%), several hundred images were available for each TP. Two cloud-masking functions in GEE were used to mask out the clouds: cloudMaskL457 and maskL8sr, which use a QA band denoted as *pixel_qa* (Fig. 1). These were implemented for the Landsat-5 and Landsat-8 data, respectively. Finally, a single cloud-free composite image was generated for each TP using the GEE median (*ee.reducer*) composite function. The function calculates the median value of all the pixels in matching bands across the image stacks and eliminated pixels with extreme value. It also removes any remaining artefacts in the imagery. Hence, each TP image represented pixels with no or minimum cloud cover (or shadow).

### Image classification

A pixel-based classification approach was used in this study. The Random Forest classification model was used (Breiman 2001). It is one of the most widely used classification algorithms in remote sensing and has also shown reliable results in wetland mapping (Amani et al. 2019; Mahdianpari et al. 2020). It is an ensemble classifier that uses a large number of decision trees that combine decisions from a set of classifiers using a voting system (Breiman 2001; Pal 2005). GEE has implemented a slightly modified version of the algorithm known as the SMILE random forest (Li 2016), which was used here. It closely resembles the original algorithm in structure but includes decision tree size regularisation through the *maxNode* and *nodeSize* parameters.

The optimum value for the two most important parameters in the random forest classifier, namely the number of trees and number of variables, was adjusted using a trial-and-error method and the square root of the number of features, respectively. A value of 20 was finally used for the number of trees. The model was trained for each TP using the training samples. Due to spectral similarities between the industrial peatlands and many residual peatland sites, most of the areas not identified as industrial extraction sites (containing bare peat on the surface and mostly non-industrial) were classified as such. To resolve this issue, boundary data from BnM was used to delineate the spatial extent of the industrial peat extraction sites on raised bogs and blanket bogs. Any bare (exposed) peat areas, outside the BnM industrial extraction sites that were classified as industrial peatlands, were merged with residual peatlands. This is due to the coarse spatial resolution of the imagery and could be improved in future studies with higher spatial resolution datasets. National-scale peatland land use maps at 30-m spatial resolution were thus created for each TP. The output maps from this analysis depict the spatial extent and temporal trend of four major land use categories on Irish peatlands over a 30-year period.

### Accuracy assessment using validation data

An overall accuracy assessment was conducted for the output maps using independent data. The maps were uploaded to the SEPAL platform (FAO 2020) which is a cloud-computing-based platform developed for the automated mapping, monitoring and assessment of land cover. There are several tools available on the platform for land cover analysis. In this study, the following tools were used; stratified area estimator–design (SAED) and stratified area estimator–analysis (SAEA). SAED was used to create a sampling point. The tool uses Eq. (1) (Cochran 2007) to calculate the overall sample size (*n*). It ensures that enough sample points (validation data) are created for each stratum (class).

1 $$n=\frac{{(\sum {W}_{i}{S}_{i})}^{2}}{{[S\widehat{(O)}]}^{2}+\left(\frac{1}{N}\right){\sum {W}_{i}{S}_{i}}^{2}}\approx {\left(\frac{(\sum {W}_{i}{Ss}_{i})}{Ss\widehat{(O)}}\right)}^{2}$$

where *n* is the total number of validation points, *N* is the number of pixels for each class in the classified raster representing the spatial unit, *S*(Ô) represents the standard error of the estimated overall accuracy, *S*_*i*_ is the standard deviation of each class (stratum), *S*_*i*_ = $\sqrt{{U}_{i}(1- {U}_{i}}$, where *U*_*i*_ is the conjectured value of the user’s accuracy for a land use class and *W*_*i*_ is the proportional area of each class *i* that is mapped (Olofsson et al. 2014). One thousand seven hundred four sample points were generated which were used for accuracy assessment (Table 3).

**Table 3:** Distribution of validation sample points for each land use derived using the SEPAL

Land use class	Sample points
Forest	219
Grassland	394
Industrial	100
Residual peatland	991
Total	1704

The labelling of validation data was carried out using Collect Earth, a desktop-based tool combining Google Earth and Google Earth Engine (Bey et al. 2016). This was conducted employing visual interpretation of high-resolution imagery in the Google Earth desktop application in both 2005 and 2019. Each point was carefully assessed by a single expert operator. For areas with low spatial resolution imagery, the temporal archive and GEE were utilised to ascertain the specific class; however, these points were less than 10% of the total. Upon completion of the labelling process, the points were further analysed in the SEPAL platform using the SAEA tool. This was used to obtain a confusion matrix and estimate the overall, producer and user accuracies. The simplest statistic is the overall accuracy which defines the proportion of locations that are correctly predicted by the model. The producer’s accuracy indicates the probability of a correctly classified reference pixel, whereas the user’s accuracy defines the probability of the pixel classified on the map agreeing with the class on the ground (Story and Congalton 1986; Congalton 1991).

## Results

**Fig. 3:**
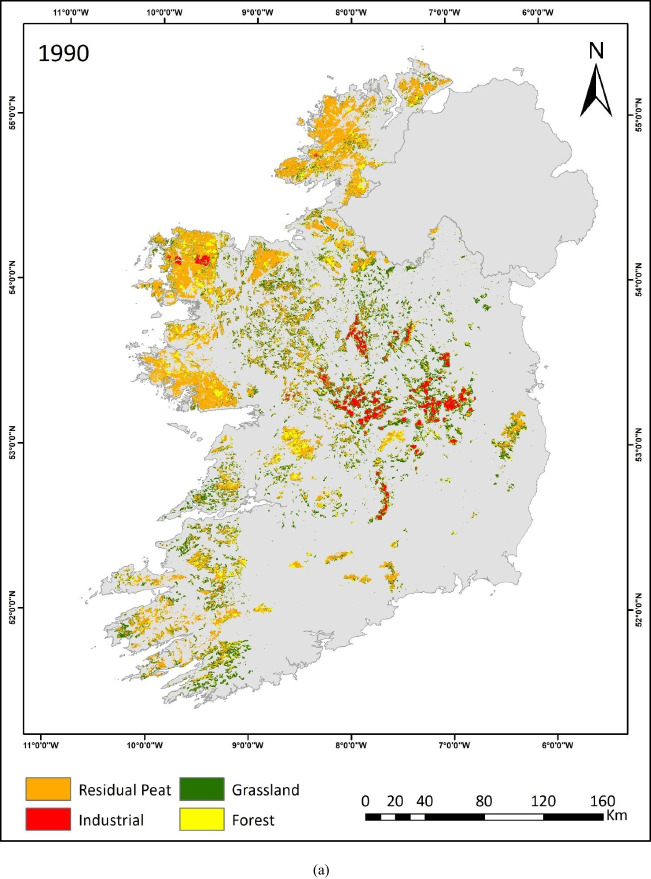

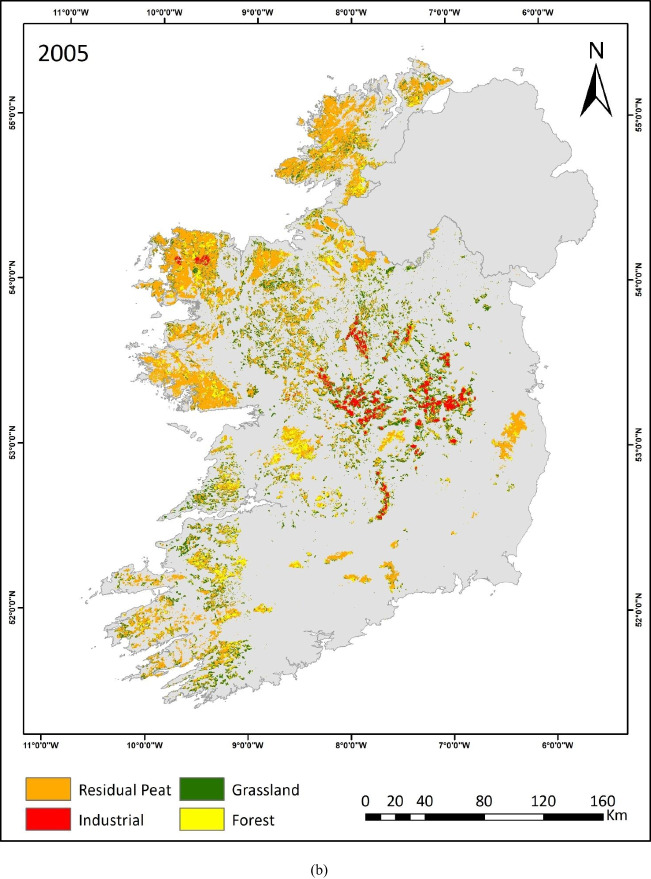

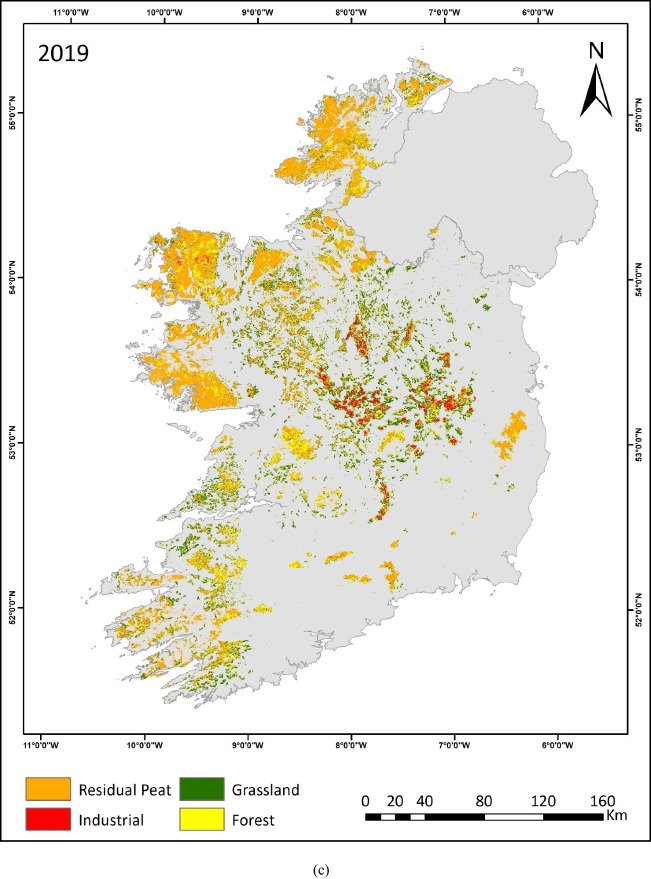
**a** Peatland Land use map 1990. **b** Peatland land use map 2005. **c** Peatland land use map 2019

This study is the first to quantify land use on raised and blanket bogs separately across Ireland over three decades. The resulting maps (depicted in Fig. 3a, b, c) provide insights into the intensity, extent and trends of land use on peatlands during this period. The prevalence of industrial peat extraction sites can be seen in the midland region, where most of the raised bogs are situated. These peatlands are transitioning into grassland and forestry, as evident in the 2019 map (Fig. 3c). Within these midland areas, grassland primarily occupies the peripheries of industrial peat extraction sites. Additionally, grassland emerges as a predominant land use category across both peatland types. Along the western seaboard’s blanket bog regions, forest and residual peatland remain prevalent.

**Fig. 4:**
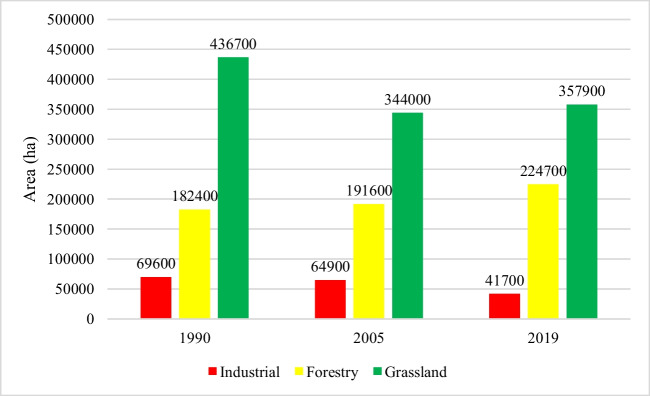
Comparison between land use areas for industrial, forest and grasslands

The spatial extent of each peatland land use category was assessed for each TP. The industrial peatland extraction sites managed by BnM have undergone a significant transition. One key indicator of this change is the steady reduction in exposed bare peat areas, which have decreased by almost 40%, from 69,600 to 41,700 ha between 1990 and 2019 (Fig. 4). Afforestation increased by 17% between 2005 and 2019 from 191,600 to 224,700 ha, respectively. Over the past 30 years, the grassland area has decreased from 436,700 to about 357,900 ha (Fig. 4).

Land use change dynamics between 1990 to 2005 and 2005 to 2019 are depicted in Tables 4 and 5, respectively. These tables show the major land use change between the four mapped land use classes. The bold values along the diagonal line in the tables indicate the land use categories that remained constant and were consequently subjected to the same land use. The cumulative area represented by these constant values is estimated to be approximately 1 Mha between 1990 and 2005, and 1.1 Mha between 2005 and 2019. This constitutes ~ 71% of the total peatland area, identified at this spatial resolution.

**Table 4:** Land use (LU) change (ha) in Irish peatlands from 1990 to 2005. The emboldened diagonal elements represent constant land use

		Land use 2005				
	LU class	Forest	Grassland	Industrial	Residual peat	Total (1990)
Land use 1990	Forest	**95,300**	21,500	700	64,900	182,400
	Grassland	40,700	**273,300**	1100	121,600	436,700
	Industrial	2300	400	**59,700**	7200	69,600
	Residual peat	53,300	48,800	3400	**665,800**	771,300
	Total (2005)	191,600	344,000	64,900	859,500	1,460,000
	Change (ha)	9200	− 9270	− 470	88,200	
	Change (%)	5	− 27	− 7	11

**Table 5:** Land use (LU) change (ha) in Irish peatlands from 2005 to 2019. The emboldened diagonal elements represent constant land use

		Land use 2019				
	LU class	Forest	Grassland	Industrial	Residual peat	Total (2005)
Land use 2005	Forest	**119,200**	22,300	300	49,800	191,600
	Grassland	34,700	**249,300**	0	60,000	344,000
	Industrial	5100	2800	**39,500**	17,500	64,900
	Residual peat	65,700	83,500	1900	**708,400**	859,500
	Total (2019)	224,700	357,900	41,700	835,700	1,460,000
	Change (ha)	33,100	13,000	− 2320	− 2380	
	Change (%)	17	4	− 36	− 3	

The other ~ 30% of the peatland area has experienced a change in land use. The largest change between 1990 and 2005 was a 27% reduction in the grassland sites due to afforestation, i.e. 40,700 ha. Afforestation showed a 5% increase in 2005 compared to 1990. The conversion of grassland to residual peatland, in some cases, could be attributed to differences in the spectral signature and is discussed in detail later. Meanwhile, the industrial peat extraction sites underwent substantial land use change between 2005 and 2019 (Fig. 3b, c). Almost one-third (36%) of these sites were converted to forest, grassland and residual peatlands. The increase in afforestation is also apparent as 34,700 ha of grasslands and 5100 ha of industrial peat extraction sites converted to forestry (Table 5).

### Raised bogs

Grassland (agriculture) has been the dominant land use in raised bogs, accounting for ~ 43% of the total area of raised bogs in 1990 and 47% in 2019 (Table 6). A smaller but significant area has been afforested, increasing from ~ 8% in 1990 to ~ 14% in 2019. Peat soil exposed through industrial peat extraction constituted ~ 12% in 1990 reducing to ~ 7% in 2019. Approximately 90% of the BnM industrial peat extraction sites occur on raised bogs, in the midlands. However, there has been a notable decrease in industrial bare peat sites, as they transition to other land use types.

**Table 6:** Land use (LU) on raised bogs over three decades

LU classes	1990		2005		2019	
	Area (ha)	%	Area (ha)	%	Area (ha)	%
Industrial	61,900	11.5	58,100	10.8	39,900	7.4
Forest	42,000	7.8	51,500	9.6	73,400	13.6
Grassland	255,900	47.5	206,900	38.4	232,800	43.2
Residual peat	179,100	33.2	222,400	41.3	192,800	35.8
Total	538,900	100	538,900	100	538,900	100

### Blanket bogs

Much less land use change can be detected in blanket bogs (Table 7) at this spatial resolution. Grasslands on peatlands have decreased from 20% in 1990 to 14% in 2019. Afforested areas have increased marginally from 16 to 17%. The residual peat area increased slightly between 1990 and 2019 from 64 to 70%. However, as with the raised bogs, the residual peat category is a catchall for many peatland land use categories that cannot be detected at this spatial resolution. The BnM industrial areas at Bellacorrick, Co. Mayo, are now captured under the residual peatland category. These industrial peat extraction sites on blanket bogs account for between 0.2 and 0.8% of the total area of BnM landholdings. However, peat extraction ceased there in 2003 and there has been a transition from industrial to forest and residual peatlands. This may depict revegetation through rehabilitation activities after the cessation of extraction in 2003 (Farrell and Doyle 2003).

**Table 7:** Land use on blanket bogs over three decades

LU classes	1990		2005		2019	
	Area (ha)	%	Area (ha)	%	Area (ha)	%
Industrial	7300	0.8	4400	0.5	1700	0.2
Forest	144,000	15.6	140,800	15.3	151,500	16.4
Grassland	181,200	19.7	143,200	15.5	126,900	13.8
Residual peat	588,600	63.9	632,700	68.7	641,000	69.6
Total	921,100	100	921,100	100	921,100	100

### Accuracy assessment

The accuracy of the map is calculated by comparing the mapped classes against the reference data. The overall accuracy was 86% and 85% for the 2005 and 2019 maps respectively (Table 8). In both the 2005 and 2019 maps, the forest class exhibited the highest user’s accuracy, with values of 95% and 88%, respectively. The producer’s accuracy, on the other hand, was highest for residual peatland class in both 2005 and 2019 maps, i.e. 95% and 93% respectively. The accuracy assessment of the 1990 map was not carried out due to the unavailability of high-resolution reference data.

**Table 8:** Accuracy assessment results for 2005 and 2019 results

	Accuracy (%)			
LU classes	2005		2019	
	Users	Producers	Users	Producers
Forest	95	60	88	74
Grassland	89	84	85	90
Industrial	86	83	82	64
Residual peatland	82	96	86	93
Overall accuracy	86		85	

## Discussion

This study seeks to address the lack of robust spatial data on land use and land use change on peatlands in Ireland. It uses freely available Landsat satellite imagery and the GEE cloud-computing platform. A novel and robust method was developed in GEE using the random forest machine learning approach to identify four peatland land use classes and to track land use dynamics over 30 years from 1989 to 2020. The use of these methods in GEE enabled the study to overcome the challenges posed by persistent cloud cover and obtain accurate wall-to-wall land use maps for Irish peatlands. Several studies have found cloud cover to be a major issue with using remote sensing in Ireland (Cawkwell et al. 2018; Connolly 2018; Walsh et al. 2021). The findings demonstrate significant changes across all four peatland land use categories examined here. The accuracy assessment results from the 2005 and 2019 datasets show a particularly good agreement between the land use maps and the reference data; however, high-resolution reference data was not available to conduct the accuracy assessment of 1990 land use results. Table 4 provides information on the direction and magnitude of changes in land use class that facilitates the evaluation of the consistency of the land use classification for that particular TP. The transition from forest to grassland observed may be linked to policy incentives for agricultural production (Donnellan et al. 2015; Renou-Wilson and Byrne 2015), whereas small changes in bare peat in industrial areas between 1990 and 2005 are consistent with the policy regarding industrial peat extraction (Bullock et al. 2012).

In TP2: 2005, grassland covers 23% (~ 342,800 ha) of the peatland area, forestry covers 13% (~ 188,800 ha) and industrial peat extraction covers 4% (~ 65,500 ha). However, a previous study by Connolly (2018) reports higher estimates for grassland and forestry areas: 437,000 ha and 337,000 ha, respectively. Similarly, the industrial peat extraction area was estimated to be ~ 50,000 ha but did not include BnM landholdings on blanket bogs. Therefore, our study estimated an accurate BnM industrial peat extraction area of 63,900 ha. The remaining 59% (~ 864,500 ha) consists of degraded remnant and cutaway peatland areas, which were not detectable in either study due to the coarse spatial resolution of the imagery. We believe that the utilisation of robust machine learning-based pixel classification techniques, coupled with cloud-free wall-to-wall coverage, as employed in our study, enables more accurate estimations of the four land use categories when compared to the previous study (Connolly 2018).

Peatlands have been under considerable land use pressure in Ireland, particularly over the past several decades with the introduction of mechanised peat extraction (Smith and Crowley 2020). There are varying spatial and temporal changes across raised and blanket bogs. Industrial peat extraction areas are being converted to grassland, forestry and residual peatlands (revegetated and abandoned), whereas grasslands and forestry, located on peatlands, can be replaced with each other. According to this pixel-based approach, approximately 30% or 400,000 ha of Ireland’s peatlands have experienced a change from one type of peatland land use to another in the past 30 years. Of the remaining 70%, about 47% were classed as residual peatlands. The land use changes that occur in those areas are relatively small scale, e.g. domestic peat extraction and are not detectable using Landsat data. However, they are extensive and should be examined using higher-resolution imagery in future studies. The remaining 23% represent land use on peatlands including industrial, forested or grassland, that have remained stable over the study period. The driving factors for the land use change detected in this study could be linked to land use policy changes related to peat mining, afforestation and agricultural production over the last thirty years.

### Industrial

**Fig. 5:**
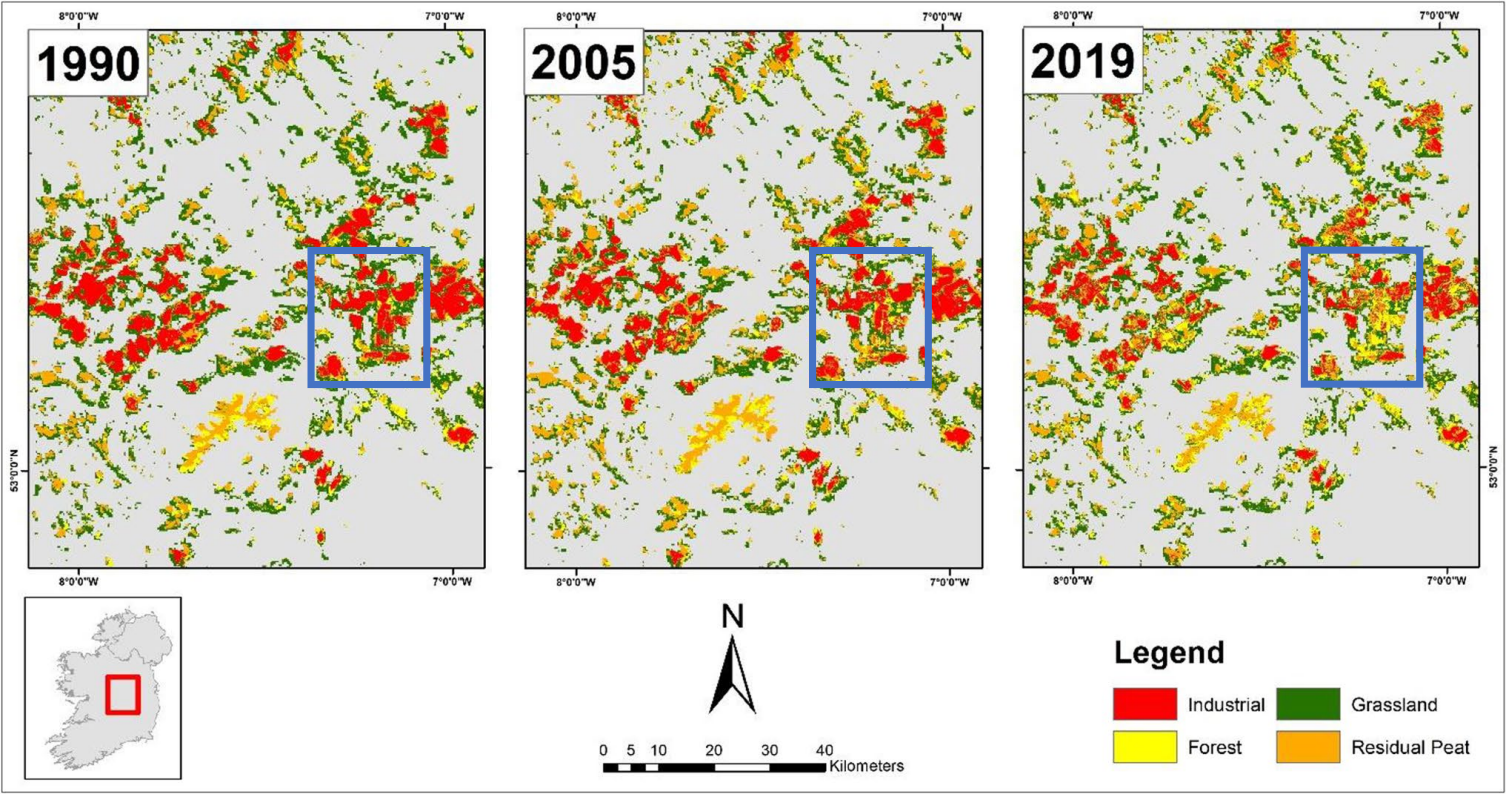
Peatland land use change in the midlands

The assessment of industrial land use was limited to the BnM landholding. The increase of afforestation on these lands has mainly occurred in the past 15 years. This could be attributed to the afforestation of former industrial peat extraction sites by BnM in collaboration with Coillte as depicted in Fig. 5. It also shows the transition from industrial peat extraction sites to grassland and residual peatland. Between 1990 and 2005, there were minor changes in the east of the midlands in the areas (outlined by the blue box in Fig. 5). However, between 2005 and 2019, there is a marked increase in forestry (yellow) and residual peat (orange) in this area. Given the relatively rapid land use change detected between 2005 and 2019, it would be prudent to track and monitor this on all industrial peatland sites using this methodology particularly, as all extraction activities on these sites officially ended in 2020 (BnM 2021).

### Forestry

While there is an overall increase in forestry on peatlands over the past three decades, there was also a sub-trend where some afforested areas were converted to residual peatlands. For example, several EU LIFE-Nature projects have the aim of the “restoration of afforested bogs to their former status” including 1976 ha of blanket bogs on the west coast and more recently the raised bogs sites in the midlands (Bullock et al. 2012; Griffin 2016). This increasing trend may be related to afforestation policy incentives offered on a national scale. These incentives began with the establishment of Coillte in 1989 (Ward 1989), a semi-governmental organisation for the management of state-owned forestry. The introduction of EU and national afforestation schemes and grants such as the farm compensatory allowance scheme in 1987, the forest premium in 1990 and more recently, the afforestation scheme 2014–2020 incentivised forestry for farmers/landowners (Government of Ireland 2022). In this study, a substantial increase in forestry on peatlands was detected in the past 15 years, mainly on raised bogs. There are further plans by BnM and Coillte, e.g. to convert 1500 ha of the former industrial area into native woodlands (BnM 2019). Therefore, this land use change trend will continue over the coming years.

### Grassland

**Fig. 6:**
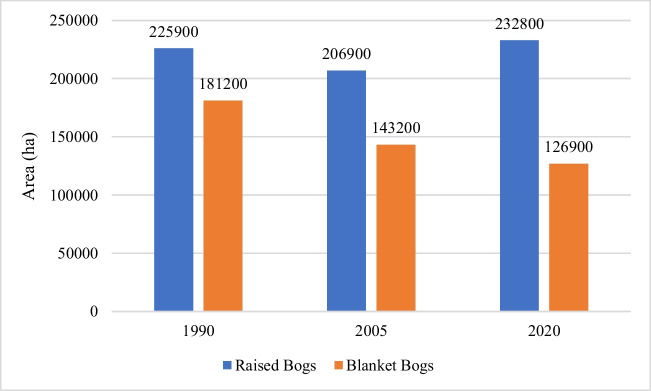
Grassland area on raised and blanket bogs

Irish peatlands have been drained and reclaimed for agricultural development for centuries (Hammond 1981; Connolly 2018). This grassland category includes several types of grassland land uses including silage, hay, grazing and pasture (Fealy et al. 2009). While the other land use categories exhibit either consistent downward or upward trends since 1990, the grassland area on peatlands in Ireland has experienced an overall decline between 1990 and 2019. However, between 2005 and 2019, an increase is observed. A more detailed examination of this phenomenon in Fig. 6 highlights a continuous decrease in grassland on blanket bogs, while grasslands on raised bogs display a decrease between 1990 and 2005, followed by an increase in 2019.

To be precise, the land use conversion analysis (Table 5) shows that between 1990 and 2005, ~ 40,700 ha of grassland on peatland have been converted to forestry. This could be attributed to afforestation incentives discussed earlier. The increase in grasslands on raised bogs between 2005 and 2019 could be explained by a national drive towards agricultural production with incentives such as the abolition of the milk quota (Donnellan et al. 2015). BnM also converted 2500 ha of industrial cutaway land to agricultural grasslands (Renou-Wilson et al. 2015). Furthermore, government grant aids in the 1980/90 and payments through the EU Common Agricultural Policy (CAP), encouraged the conversion of large areas of peatland (mostly upland bogs) to grazing lands (Bullock et al. 2012).

### Residual peatland

The residual peatland class comprises a range of small-scale land use categories that make up a spatially complex and heterogenous landscape (Connolly 2018). These land use categories include domestic peat extraction, rewetted and rehabilitated areas, natural degradation and bare peat which can be difficult to discriminate within the Landsat imagery. Consequently, the spectral signature variations are likely the result of land use and revegetation associated with the rehabilitation or abandonment of these lands. This leads to a shift from one class to another within a particular TP, followed by a return to the same class. An example of this is a change from grassland to residual peatland and then back to grassland. Sites that are going through rehabilitation such as the former BnM industrial site at Bellacorrick, Co. Mayo have predominantly reverted to poor fen and scrub with embryonic peat-forming conditions and thus converted to the residual peatland category (Farrell and Doyle 2003). The cutover and remnant peatland (high bog) areas present a dynamic landscape and detailed habitat (area of occupancy) mapping using high-resolution imagery is essential (Álvarez-Martínez et al. 2018b; Ingle et al. 2023). COPERNICUS Sentinel-2 or other higher-resolution imagery could be used for this purpose, but this was beyond the scope of this study. Future work could also include additional TPs and the analysis of the spectral signature over time.

Overall, the general spatial distribution and temporal trend of the four main land use classes show that peatland industrial areas, which are present mostly on raised bogs, are converting to residual peatland, forestry and grassland. Although residual peatlands possibly show the rehabilitation, restoration and rewetting efforts being implemented. Furthermore, the use of blanket bogs for afforestation activities will also lead to ecosystem degradation and increasing peatland emissions (Jovani-Sancho et al. 2021). Degradation could be further exacerbated by climate change. For instance, periods of dry weather might render these ecosystems more susceptible to wildfires, and episodes of drought followed by heavy precipitation could increase their vulnerability to landslides (Connolly et al. 2021).

The overall accuracy for the maps in both 2005 and 2019 is 86% and 85%. However, more detailed information can be obtained by looking at the user and producer accuracy. The user accuracy for forestry and grassland is the highest in the 2005 (86%) and 2019 (85%) maps. The accuracy of the residual peat class is the lowest, primarily because this category is an amalgamation of multiple small-scale land uses. This is a limitation of using medium spatial resolution data (30 m). The industrial class also has lower accuracy. This class represents the exposed peat areas within the BnM landholdings. However, the peripheries of BnM sites still have domestic peat extraction activities ongoing which result in exposed peat. This is one cause of confusion between industrial peat extraction sites with residual peatlands, resulting in lower accuracy for both classes. Future work could use high-resolution satellite imagery and very high-resolution aerial imagery to improve the land use maps, and map residual peatland class in further detail. This includes domestic peat extraction and rehabilitation, restoration and rewetting sites. The use of Landsat (medium resolution) for the hard classification of large areas may introduce uncertainties (Álvarez-Martínez et al. 2010). For example, in heterogeneous land use classes such as the residual peatlands and grassland in this study, the allocation of pixels to specific land use classes may cause uncertainty (Bradley and Mustard 2005). This could introduce errors in classification results and eventually the confusion matrix. The elimination of these uncertainties using the methods proposed by Álvarez-Martínez et al. (2010) could lead to a more refined accuracy assessment and a more efficient analysis of the temporal evolution of changes. Additionally, this study uses an existing peatland map (DIPMv2) for the delineation of peatland areas, although it is the latest and most reliable map of peatlands in Ireland with an overall accuracy of 88%. It underestimates the extent of peatlands with areas of less than 7ha (Connolly and Holden 2009). Therefore, this study can be expanded to these missing areas if that peat map is updated. Nevertheless, our study is an essential first step towards understanding peatland land use and offers a significant perspective and valuable insights into the dynamics of land use change on peatlands in Ireland.

While higher spatial resolution remote sensing data is available, this methodology outputs maps of the major land use categories on peatlands providing valuable information on how they are changing through space and time. It could be a useful dataset for the recently announced “Land Use Review” by the Government of Ireland (2023). This methodology could be used to identify hotspots of land use change to support policy-related decision-making, aid Nation Inventory Reporting, promote sustainable land use practices and implement the principles and actions outlined in the National Peatland Strategy of Ireland. Additionally, these maps could also be used to refine estimates of GHG emissions from peatlands using Intergovernmental Panel on Climate Change (IPCC 2003) methodologies. The method used here is robust and could be applied to track and monitor peatland land use and land use change over time both in Ireland and across the globe.

## Conclusion

This study presents the first long-term spatiotemporal assessment of peatland land use and land use change for Ireland. It is conducted over a 30-year period from 1989 to 2020. The spatial extent of four major peatland land use categories (grassland, forestry, industrial peat extraction and residual peatlands) are quantified, for both blanket and raised bogs. The methodology integrates the DIPMv2 to delimit peatlands within the GEE cloud-computing platform. The random forest machine learning algorithm is combined with Landsat data to classify the major land use classes. The results indicate that at least 30% of peatlands in Ireland have undergone major land use change in the last 30 years. The results clearly show the conversion of industrial peatlands (BnM owned) as extraction ceases and the land is converted to several other land uses including rewetted areas. The forestry area has also increased, and grassland is one of the largest land use classes on peatlands in Ireland. The methodology also showcases how GEE can be used to overcome issues such as persistent cloud cover to deliver a national-scale assessment of land use change on peatlands and identify hotspots of change. Future studies should use higher-resolution imagery that can then be applied to examine these areas in detail, particularly the residual peatland class where change is occurring but cannot be detected with Landsat data. This robust method could be used to generate peatland land use-specific maps for peatland areas across the globe using the available global and regional peatland extent maps.

## Data Availability

The data that support the findings of this study are openly available in the Open Science Framework data respository at 10.17605/OSF.IO/V4NSQ.

## References

[CR1] Álvarez-Martínez JM, Stoorvogel JJ, Suárez-Seoane S, de Luis Calabuig E (2010). Uncertainty analysis as a tool for refining land dynamics modelling on changing landscapes: a case study in a Spanish Natural Park. Landsc Ecol.

[CR2] Álvarez-Martínez JM, Silió-Calzada A, Barquín J (2018). Can training data counteract topographic effects in supervised image classification? A sensitivity analysis in the Cantabrian Mountains (Spain). Int J Remote Sens.

[CR3] Álvarez-Martínez JM, Jiménez-Alfaro B, Barquín J, Ondiviela B, Recio M (2018). Modelling the area of occupancy of habitat types with remote sensing. Methods Ecol Evol.

[CR4] Amani M, Brisco B, Afshar M, Mirmazloumi SM, Mahdavi S (2019). A generalized supervised classification scheme to produce provincial wetland inventory maps: an application of Google Earth Engine for big geo data processing. Big Earth Data.

[CR5] Bey A, Sánchez-Paus Díaz A, Maniatis D, Marchi G, Mollicone D (2016). Collect earth: land use and land cover assessment through augmented visual interpretation. Remote Sens.

[CR6] BnM (2019) Bord na Móna and Coillte collaborate to transform 1500 hectares into native woodland - Bord Na Mona. In: Bord na Móna. https://www.bordnamona.ie/bord-na-mona-and-coillte-collaborate-to-transform-1500-hectares-into-native-woodland/. Accessed 10 Aug 2022

[CR7] BnM (2021) Bord na Móna announce formal end to all peat harvesting on its lands. In: Bord na Móna. https://www.bordnamona.ie/bord-na-mona-announce-formal-end-to-all-peat-harvesting-on-its-lands/. Accessed 18 Aug 2023

[CR8] Bossard M, Feranec J, Otahel J (2000) CORINE land cover technical guide: Addendum 2000. European Environment Agency Copenhagen. https://www.eea.europa.eu/publications/COR0-landcover. Accessed 18 Aug 2023

[CR9] Bradley BA, Mustard JF (2005). Identifying land cover variability distinct from land cover change: cheatgrass in the Great Basin. Remote Sens Environ.

[CR10] Breiman L (2001). Random forests. Mach Learn.

[CR11] Bullock CH, Collier MJ, Convery F (2012). Peatlands, their economic value and priorities for their future management - the example of Ireland. Land Use Policy.

[CR12] Cawkwell F, Dwyer N, Scarrott R (2010) Industrialised peat extraction scoping project. In: UCC, Cork. https://www.friendsoftheirishenvironment.org/images/peat/gis/peat_cat_final_report_nov2010.pdf. Accessed 18 Aug 2023

[CR13] Cawkwell F, Raab C, Barrett B, Green S, Finn J (2018) TaLAM: mapping land cover in lowlands and uplands with satellite imagery. https://www.epa.ie/publications/research/waste/Research_Report_254.pdf. Accessed 18 Aug 2023

[CR14] Cochran WG (2007). Sampling techniques.

[CR15] Congalton RG (1991). A review of assessing the accuracy of classifications of remotely sensed data. Remote Sens Environ.

[CR16] Connolly J (2018). Mapping land use on Irish peatlands using medium resolution satellite imagery. Irish Geogr.

[CR17] Connolly J, Holden NM (2009). Mapping peat soils in Ireland: updating the derived Irish peat map. Irish Geogr.

[CR18] Connolly J, Holden NM (2011). Object oriented classification of disturbance on raised bogs in the Irish Midlands using medium- and high-resolution satellite imagery. Irish Geogr.

[CR19] Connolly J, Holohan E, Bourke M, Cruz C, Farrell C et al (2021) Characterisation of the 2020 Drumkeeran peat landslide: a large peat slide in Ireland. In: EGU General Assembly Conference Abstracts, pp EGU21–13007. 10.5194/egusphere-egu21-13007

[CR20] Czapiewski S, Szumińska D (2021). An overview of remote sensing data applications in peatland research based on works from the period 2010–2021. Land.

[CR21] Davies H, Forster C (2014) Ireland’s Forestry Programme 2014–2020. ADAS UK, Leeds. https://irishriverproject.com/wp-content/uploads/2022/03/forestryprogramme20142020naturaimpactstatement230215.pdf . Accessed 18 Aug 2023

[CR22] Donnellan T, Hennessy T, Thorne F (2015) The end of the quota era: a history of the dairy sector and its future prospects. https://www.teagasc.ie/media/website/publications/2015/End_of_the_Quota_Era_final.pdf. Accessed 18 Aug 2023

[CR23] Dronova I (2015). Object-based image analysis in wetland research: a review. Remote Sens.

[CR24] EU (2018). Regulation (EU) 2018/841 of the European Parliament and of the Council of 30 May 2018 on the inclusion of greenhouse gas emissions and removals from land use, land use change and forestry in the 2030 climate and energy framework, and amending Regulation. Off J Eur Union.

[CR25] EU (2022) Regulation of the European Parliament and of the council on nature restoration. https://www.europarl.europa.eu/doceo/document/A-9-2023-0220_EN.html. Accessed 18 Aug 2023

[CR26] FAO (2020) SEPAL Repository. In: Available online. https://github.com/openforis/sepal/. Accessed 31 Mar 2021

[CR27] Farrell CA, Doyle GJ (2003). Rehabilitation of industrial cutaway Atlantic blanket bog in County Mayo, north-west Ireland. Wetl Ecol Manag.

[CR28] Fealy R, Green S, Loftus M, Meehan R, Radford T et al (2009) Teagasc/EPA soil and subsoils mapping project. In: Final Rep. https://t-stor.teagasc.ie/handle/11019/361. Accessed 18 Aug 2023

[CR29] Feehan J, O’Donovan G (1996). The Bogs of Ireland.

[CR30] Fluet-Chouinard E, Stocker BD, Zhang Z, Malhotra A, Melton JR (2023). Extensive global wetland loss over the past three centuries. Nature.

[CR31] Foga S, Scaramuzza PL, Guo S, Zhu Z, Dilley RD (2017). Cloud detection algorithm comparison and validation for operational Landsat data products. Remote Sens Environ.

[CR32] Foss PJ (2007) Study of the extent and conservation status of springs, fens and flushes in Ireland 2007. In: Natl. Park. Wildl. Serv. Irel. https://www.npws.ie/sites/default/files/publications/pdf/Foss_%26_Crushell_2007_Fen_report.pdf. Accessed 18 Aug 2023

[CR33] Gorelick N, Hancher M, Dixon M, Ilyushchenko S, Thau D (2017). Google Earth Engine: planetary-scale geospatial analysis for everyone. Remote Sens Environ.

[CR34] Gorham E (1991). Northern peatlands: role in the carbon cycle and probable responses to climatic warming. Ecol Appl.

[CR35] Government of Ireland (2020) Cabinet approves €108 million funding for groundbreaking Bord na Móna bog rehabilitation plan. https://www.gov.ie/en/press-release/2aae1-cabinet-approves-108m-funding-for-groundbreaking-bord-na-mona-bog-rehabilitation-plan-minister-ryan-also-announces-that-47-more-projects-in-the-midlands-totalling-278m-are-approved-under-the-just-transition-fund/. Accessed 29 Oct 2021

[CR36] Government of Ireland (2021) Ireland will now report greenhouse gas emissions and removals from managed wetlands (and including bogs) as part of progress towards EU greenhouse gas targets. https://www.gov.ie/en/press-release/005d3-ireland-will-now-report-greenhouse-gas-emissions-and-removals-from-managed-wetlands-and-including-bogs-as-part-of-progress-towards-eu-greenhouse-gas-targets/. Accessed 29 Oct 2021

[CR37] Government of Ireland (2022) Forestry grants and schemes. https://www.gov.ie/en/publication/e384e-forestry-grants-and-schemes/. Accessed 21 Aug 2023

[CR38] Government of Ireland (2023) Land use review – phase 1. https://www.gov.ie/en/publication/f272c-land-use-review-phase-1/. Accessed 21 Aug 2023

[CR39] Griffin E (2016) Ecosystem Services Coillte ’ s progress in the provision of public goods across its estate. In: Irish For. https://journal.societyofirishforesters.ie/index.php/forestry/article/view/10855. Accessed 18 Aug 2023

[CR40] Hammond RF (1981) The Peatlands of Ireland, 2nd edn. An Foras Taluntais, Dublin

[CR41] Hastie A, Honorio Coronado EN, Reyna J, Mitchard ETA, Åkesson CM (2022). Risks to carbon storage from land-use change revealed by peat thickness maps of Peru. Nat Geosci.

[CR42] Ingle R, Habib W, Connolly J, McCorry M, Barry S et al (2023) Upscaling methane fluxes from peatlands across a drainage gradient in Ireland using PlanetScope imagery and machine learning tools. Sci Rep 13. 10.1038/s41598-023-38470-6 10.1038/s41598-023-38470-6 PMC10368722 37491422

[CR43] IPCC (2003) IPCC (Intergovernmental Panel on Climate Change) Good practice guidance for land use, land-use change and forestry. http://www.ipcc-nggip.iges.or.jp. Accessed 1 Sep 2022

[CR44] Jackson RB, Lajtha K, Crow SE, Hugelius G, Kramer MG (2017). The ecology of soil carbon: pools, vulnerabilities, and biotic and abiotic controls. Annu Rev Ecol Evol Syst.

[CR45] Jovani-Sancho AJ, Cummins T, Byrne KA (2021). Soil carbon balance of afforested peatlands in the maritime temperate climatic zone. Glob Chang Biol.

[CR46] Krankina ON, Pflugmacher D, Friedl M, Cohen WB, Nelson P (2008). Meeting the challenge of mapping peatlands with remotely sensed data. Biogeosciences.

[CR47] Li H (2016) Smile - Statistical Machine Intelligence and Learning Engine. https://haifengl.github.io/classification.html#random-forest. Accessed 19 Mar 2022

[CR48] Limpens J, Berendse F, Blodau C, Canadell JG, Freeman C (2008). Peatlands and the carbon cycle: from local processes to global implications–a synthesis. Biogeosciences.

[CR49] Loisel J, Gallego-Sala AV, Amesbury MJ, Magnan G, Anshari G (2021). Expert assessment of future vulnerability of the global peatland carbon sink. Nat Clim Chang.

[CR50] Loveland TR, Reed BC, Brown JF, Ohlen DO, Zhu Z (2000). Development of a global land cover characteristics database and IGBP DISCover from 1 km AVHRR data. Int J Remote Sens.

[CR51] Lu D, Weng Q (2007). A survey of image classification methods and techniques for improving classification performance. Int J Remote Sens.

[CR52] Lukacz PM (2022) Data capitalism, Microsoft’s planetary computer, and the biodiversity informatics community. In: International conference on information. Springer, pp 355–369. 10.1007/978-3-030-96957-8_31

[CR53] Mahdianpari M, Salehi B, Mohammadimanesh F, Brisco B, Homayouni S et al (2020) Big data for a big country: the first generation of Canadian wetland inventory map at a spatial resolution of 10-m using Sentinel-1 and Sentinel-2 data on the Google Earth Engine cloud computing platform: Mégadonnées pour un grand pays: La première carte. Can J Remote Sens 1–19. 10.1080/07038992.2019.1711366

[CR54] Malone S, O’Connell C (2009) Ireland’s peatland conservation action plan. https://www.ipcc.ie/a-to-z-peatlands/irelands-peatland-conservation-action-plan/. Accessed 18 Aug 2023

[CR55] Masek JG, Vermote EF, Saleous NE, Wolfe R, Hall FG (2006). A Landsat surface reflectance dataset for North America, 1990–2000. IEEE Geosci Remote Sens Lett.

[CR56] Montanarella L, Jones RJA, Hiederer R (2006). The distribution of peatland in Europe. Mires Peat.

[CR57] Olofsson P, Foody GM, Herold M, Stehman SV, Woodcock CE (2014). Good practices for estimating area and assessing accuracy of land change. Remote Sens Environ.

[CR58] Pal M (2005). Random forest classifier for remote sensing classification. Int J Remote Sens.

[CR59] Palviainen M, Peltomaa E, Laurén A, Kinnunen N, Ojala A et al (2021) Water quality and the biodegradability of dissolved organic carbon in drained boreal peatland under different forest harvesting intensities. Sci Total Environ 150919. 10.1016/j.scitotenv.2021.150919 10.1016/j.scitotenv.2021.150919 34653471

[CR60] Peacock M, Audet J, Bastviken D, Futter MN, Gauci V (2021). Global importance of methane emissions from drainage ditches and canals. Environ Res Lett.

[CR61] Pflugmacher D, Krankina ON, Cohen WB (2007). Satellite-based peatland mapping: potential of the MODIS sensor. Glob Planet Change.

[CR62] Regan S, Flynn R, Gill L, Naughton O, Johnston P (2019). Impacts of groundwater drainage on peatland subsidence and its ecological implications on an Atlantic raised bog. Water Resour Res.

[CR63] Renou-Wilson F, Byrne KA (2015) Irish peatland forests: lessons from the past and pathways to a sustainable future. Restor Boreal Temp For Second Ed 321–335. 10.1201/b18809

[CR64] Ribeiro K, Pacheco FS, Ferreira JW, de Sousa-Neto ER, Hastie A (2021). Tropical peatlands and their contribution to the global carbon cycle and climate change. Glob Chang Biol.

[CR65] Richards JA (2013). Remote sensing digital image analysis: an introduction. Remote Sens Digit Image Anal an Introd.

[CR66] Smith GF, Crowley W (2020) The habitats of cutover raised bog Irish Wildlife Manuals 128. In: NPWS. https://www.npws.ie/sites/default/files/publications/pdf/IWM128.pdf. Accessed 18 Aug 2023

[CR67] Story M, Congalton RG (1986). Accuracy assessment: a user’s perspective. Photogramm Eng Remote Sensing.

[CR68] Tanneberger F, Tegetmeyer C, Busse S, Barthelmes A, Shumka S (2017). The peatland map of Europe. Mires Peat.

[CR69] Tanneberger F, Moen A, Barthelmes A, Lewis E, Miles L (2021). Mires in Europe—regional diversity, condition and protection. Diversity.

[CR70] Tiemeyer B, AlbiacBorraz E, Augustin J, Bechtold M, Beetz S (2016). High emissions of greenhouse gases from grasslands on peat and other organic soils. Glob Chang Biol.

[CR71] Tolnai M, Nagy JG, Bakó G (2016). Spatiotemporal distribution of Landsat imagery of Europe using cloud cover-weighted metadata. J Maps.

[CR72] Tubiello FN, Biancalani R, Salvatore M, Rossi S, Conchedda G (2016). A worldwide assessment of greenhouse gas emissions from drained organic soils. Sustain.

[CR73] Turetsky MR, Benscoter B, Page SE, Rein G, Van Der Werf GR (2015). Global vulnerability of peatlands to fire and carbon loss. Nat Geosci.

[CR74] UN (1992) United Nations Framework Convention. https://heinonline.org/HOL/P?h=hein.journals/reel1&i=263. Accessed 18 Aug 2023

[CR75] United Nations Environment Programme (2022) Global peatlands assessment: the state of the world’s peatlands. https://www.unep.org/resources/global-peatlands-assessment-2022. Accessed 18 Aug 2023

[CR76] USGS (2016) Landsat 5. https://www.usgs.gov/core-science-systems/nli/landsat/landsat-5?qt-science_support_page_related_con=0#. Accessed 23 Feb 2021

[CR77] Vermote E, Justice C, Claverie M, Franch B (2016). Preliminary analysis of the performance of the Landsat 8/OLI land surface reflectance product. Remote Sens Environ.

[CR78] Waddington JM, Rotenberg PA, Warren FJ (2001). Peat CO2 production in a natural and cutover peatland: implications for restoration. Biogeochemistry.

[CR79] Walsh E, Bessardon G, Gleeson E, Ulmas P (2021). Using machine learning to produce a very high resolution land-cover map for Ireland. Adv Sci Res.

[CR80] Ward D (1989) From then to now-recent developments in Irish forestry. In: Irish For. https://journal.societyofirishforesters.ie/index.php/forestry/article/view/9669. Accessed 18 Aug 2023

[CR81] Wilson D, Dixon SD, Artz RRE, Smith TEL, Evans CD (2015). Derivation of greenhouse gas emission factors for peatlands managed for extraction in the Republic of Ireland and the United Kingdom. Biogeosciences.

[CR82] Woodcock CE, Loveland TR, Herold M, Bauer ME (2020). Transitioning from change detection to monitoring with remote sensing: a paradigm shift. Remote Sens Environ.

[CR83] Xu J, Morris PJ, Liu J, Holden J (2018). PEATMAP: refining estimates of global peatland distribution based on a meta-analysis. CATENA.

[CR84] Yu Z, Beilman DW, Frolking S, MacDonald GM, Roulet NT (2011). Peatlands and their role in the global carbon cycle. Eos Trans Am Geophys Union.

